# Long-term voluntary exercise partially reverses obesity-associated transcriptomic remodeling in brown adipose tissue

**DOI:** 10.1016/j.metop.2026.100504

**Published:** 2026-10-06

**Authors:** Takuya Sakurai, Eriko Nozaki, Tetsuya Iwawa

**Affiliations:** a Department of Chemistry, Kyorin University School of Medicine, 6-20-2 Shinkawa, Mitaka, Tokyo, 181-8611, Japan; b Core Laboratory for Proteomics and Genomics, Kyorin University School of Medicine, 6-20-2 Shinkawa, Mitaka, Tokyo, 181-8611, Japan; c Faculty of Health and Sport Science, Doshisha University, 1-3 Tatara Miyakodani, Kyotanabe, Kyoto, 610-0394, Japan

**Keywords:** Brown adipose tissue, High-fat diet, Voluntary exercise, Transcriptome, Inflammation, Metabolic remodeling

## Abstract

**Background:**

Although exercise may counteract some changes caused by obesity-induced functional and molecular remodeling of brown adipose tissue (BAT), the extent to which long-term voluntary exercise reverses obesity-associated BAT transcriptional changes remains unclear.

**Methods:**

Male C57BL/6J mice were assigned to the control, high-fat diet (HFD), or HFD plus voluntary exercise (HFD + VE) groups for four months (n = 4 per group). Interscapular BAT transcriptomic profiles were analyzed using the Clariom S Mouse Array. Differential expression was explored using eBayes ANOVA, and transcript clusters with an absolute fold change >2, a nominal P value < 0.05, and a mean log2 signal intensity ≥8 in the group with higher expression were selected for exploratory analyses. FDR-adjusted P values were additionally examined to account for multiple testing. Gene Ontology enrichment analysis was performed using DAVID.

**Results:**

Using the prespecified exploratory criteria (absolute fold change >2, nominal P < 0.05, and mean log2 signal intensity ≥8 in the group with higher expression), 579 transcript clusters differed between the control and HFD groups. In the HFD versus HFD + VE comparison, 89 transcript clusters met these exploratory criteria; 74 showed lower expression and 15 showed higher expression with VE. None of the 89 transcript clusters remained significant at FDR <0.05 after genome-wide multiple-testing correction. GO enrichment analysis nevertheless identified coordinated immune/inflammatory and muscle contraction-related biological processes, several of which remained significant after FDR correction for GO-term testing.

**Conclusions:**

Long-term voluntary exercise was associated with transcriptomic changes that opposed a subset of HFD-associated alterations, particularly immune and inflammatory signatures. A restricted contractile/Ca2+-related transcriptional signature was also identified, although the transcriptomic data alone do not establish altered Ca2+ signaling. These exploratory findings highlight the need for further investigation of the cellular and signaling mechanisms underlying exercise-associated BAT remodeling.

## 1 Introduction

Obesity is characterized by profound structural and functional adipose tissue remodeling and is a major risk factor for metabolic disorders like type 2 diabetes and cardiovascular disease [1,2]. Exercise is effective in preventing and improving obesity-associated metabolic dysfunction [3,4]. The metabolic benefits of exercise are investigated extensively in skeletal muscle and white adipose tissue (WAT), including exercise-associated remodeling of the adipose tissue immune microenvironment [[3], [4], [5]]. However, mounting evidence suggests that brown adipose tissue (BAT) may also contribute to exercise-induced metabolic adaptations.

WAT primarily stores excess energy as triglycerides and releases fatty acids depending on metabolic demand, while BAT mainly involves energy expenditure because of its high mitochondrial content and thermogenic capacity. BAT, which is strongly activated by sympathetic stimulation, particularly during exposure to cold, contributes to whole-body energy expenditure [6]. Although previously considered metabolically relevant mainly in small mammals and human infants, adult humans have metabolically active BAT, and its activity is inversely associated with adiposity [[7], [8], [9]]. Consequently, there is considerable interest in BAT as a potential target in preventing and treating obesity and related metabolic disorders.

The morphological and metabolic characteristics of BAT are substantially altered by obesity. Chronic exposure to excess nutrients causes lipid accumulation, impaired mitochondrial function and oxidative metabolism, and inflammatory remodeling of BAT [10,11], which may compromise its metabolic properties and contribute to systemic metabolic dysfunction. Exercise may counteract obesity-associated adipose tissue changes, and exercise-associated changes in BAT metabolism, vascularization, and inflammatory status have been reported [12,13]. Moreover, transcriptomic studies have identified exercise-responsive pathways in BAT under obese conditions [13]. However, it is not clear to what extent long-term voluntary exercise (VE) can reverse obesity-associated transcriptional changes toward a non-obese state.

Here, we sought to identify genes and biological processes altered by a high-fat diet (HFD) and subsequently changed in the opposite direction with exercise, which may provide insights into the molecular characteristics of exercise-associated BAT remodeling.

We investigated the effects of chronic HFD and voluntary VE on the transcriptomic profile of mouse interscapular BAT. Gene expression analysis was used to identify the genes and biological processes altered by an HFD and then modified by VE. We also identified HFD-responsive transcript clusters that changed in the opposite direction with VE. These analyses aimed to characterize obesity-associated transcriptional remodeling of BAT and determine how VE modifies these alterations. We hypothesized that long-term VE would oppose a subset of HFD-associated transcriptional alterations in BAT, particularly those related to metabolic and inflammatory processes.

## 2 Materials and methods

### 2.1 Animal care and VE

Six-week-old male C57BL/6J mice (Sankyo Labo Service Corporation, Tokyo, Japan) were individually housed throughout the experimental period at 23 °C under a 12:12-h light–dark cycle. The mice were randomly divided into the control (C) group (mice fed normal chow for four months), the HFD group (mice on an HFD for four months), and the HFD + VE group (mice fed an HFD for four months with access to voluntary wheel running) [14]. The HFD contained 60.7% fat, 17.9% protein, and 21.4% nitrogen-free extract (HFD-60; Oriental Yeast Co., Ltd., Tokyo, Japan).

Mice in the HFD + VE group were provided access to a running wheel (Melquest Ltd., Toyama, Japan) equipped with a permanent magnetic switch connected to a digital wheel revolution counter that recorded daily total wheel revolutions. The daily running distance was calculated by multiplying the number of revolutions by the circumference of the wheel. Each group consisted of four mice (n = 4 per group). All animal experiments were approved by the Experimental Animal Ethics Committee of Kyorin University School of Medicine (Approval No. 76) on April 1, 2018, and were conducted in accordance with institutional guidelines.

### 2.2 BAT collection and RNA extraction

Mice were sacrificed at the end of the experiment period, followed by rapid interscapular BAT excision and careful removal of surrounding skeletal muscle and connective tissue to minimize contamination. The samples were processed immediately or stored at −80 °C until analysis. Total RNA was extracted from BAT using ISOGEN (Nippon Gene, Tokyo, Japan) following the manufacturer's instructions, and its concentration and purity were determined using a NanoDrop spectrophotometer (Thermo Fisher Scientific, Waltham, MA, USA). RNA integrity was assessed by electrophoretic analysis using a TapeStation system (Agilent Technologies, Santa Clara, CA, USA). Only RNA samples with an RNA integrity number equivalent (RINe) ≥7.0 were considered to meet the quality criterion for microarray analysis. All samples included in the final microarray analysis met this criterion.

### 2.3 Microarray analysis

Genome-wide gene expression profiling was performed using the Clariom S Mouse Array (Thermo Fisher Scientific), and microarray analysis was conducted according to the manufacturer's protocol. Raw intensity data were processed using Transcriptome Analysis Console (TAC; Thermo Fisher Scientific) and normalized using the signal space transformation-robust multi-array average method. Gene expression values were expressed as log2-transformed signal intensities. Differential expression testing was performed in TAC using the eBayes ANOVA method. The final microarray analysis included four biological samples from each group (n = 4 per group), and no samples were excluded from the analysis. Array-level quality control was assessed using the Sample QC functions in TAC. All 12 arrays passed the labeling control, hybridization control, and positive-versus-negative area-under-the-curve (AUC) thresholds. Principal component analysis (PCA) was additionally inspected to assess overall sample distribution, and no samples were excluded on the basis of array-level quality control. The 12 samples were processed in four independent experimental sets performed on separate days, with each set containing one sample from each group (C, HFD, and HFD + VE). Thus, experimental processing day was balanced across the three groups. No explicit batch-effect correction was applied in the statistical model. Transcript clusters were mapped to corresponding gene symbols using the Clariom S Mouse Array annotation provided in Transcriptome Analysis Console (TAC; Thermo Fisher Scientific). Nominal P values were used for the prespecified exploratory transcript selection, and false discovery rate (FDR)-adjusted P values were additionally examined to account for multiple testing. For the exploratory analyses, transcript clusters with an absolute fold change >2, a nominal P value < 0.05, and a mean log2 signal intensity ≥8 in the group with higher expression were selected. The expression-intensity threshold was used to restrict the analyses to transcript clusters with reliably detectable expression.

To characterize HFD-associated transcriptional changes, expression profiles were first compared between the C and HFD groups. Transcript clusters meeting the exploratory criteria described above were classified according to the direction of the expression change as HFD-upregulated or HFD-downregulated transcript clusters.

Next, expression profiles were compared between the HFD and HFD + VE groups to characterize VE-associated transcriptional changes under HFD conditions. Transcript clusters meeting the same exploratory criteria were classified as showing higher or lower expression with VE and were analyzed separately to characterize the biological processes associated with each direction of the exercise response.

### 2.4 Gene Ontology (GO) enrichment analysis

GO enrichment analysis was performed using the Database for Annotation, Visualization and Integrated Discovery (DAVID; accessed August 6, 2026) [15]. GO biological process terms were used to characterize the biological processes associated with the differentially expressed genes.

In the comparison of the C versus HFD groups, HFD-upregulated and HFD-downregulated genes were analyzed separately to determine the biological processes associated with the direction of the obesity-associated transcriptional response.

In the comparison of HFD versus HFD + VE, transcript clusters meeting the prespecified nominal P-value, fold-change, and expression-intensity criteria and showing higher or lower expression with VE were analyzed separately. GO enrichment was considered exploratory on the basis of nominal enrichment P values, while FDR-adjusted enrichment P values were additionally reported to account for multiple GO-term testing. Enriched GO biological process terms were ranked by nominal statistical significance and visualized as horizontal bar plots using -log10 (P-value).

Transcript clusters showing opposite-direction changes between the C versus HFD and HFD versus HFD + VE comparisons, identified as described below, were also subjected to GO biological process enrichment analysis to characterize biological processes represented in these coordinated transcriptional shifts.

### 2.5 Identification of HFD-responsive transcript clusters showing opposite-direction changes with VE

Gene expression profiles were compared across the three experimental groups to identify HFD-responsive transcript clusters that changed in the opposite direction with VE. Transcript clusters were included when they met the prespecified exploratory criteria (absolute fold change >2, nominal P < 0.05, and mean log2 signal intensity ≥8 in the group with higher expression) in both the C versus HFD and HFD versus HFD + VE comparisons and when the direction of change in the HFD versus HFD + VE comparison was opposite to that in the C versus HFD comparison. A non-significant C versus HFD + VE comparison was not used as a criterion because lack of statistical significance does not establish equivalence or restoration to control levels.

The resulting transcript clusters were separated into those increased by HFD and decreased with VE and those decreased by HFD and increased with VE. These two sets were analyzed separately using DAVID to identify GO biological processes associated with the opposite-direction transcriptional shifts.

### 2.6 Assessment of skeletal muscle-associated gene expression

Because interscapular BAT is anatomically adjacent to skeletal muscles and GO enrichment analysis revealed that VE-altered genes were enriched for muscle contraction-related biological processes, we evaluated the expression of representative myogenic and skeletal muscle-associated genes in the microarray dataset.

Canonical myogenic regulatory factors, including myogenic differentiation 1, myogenin, myogenic factor 5 (*Myf5*), myogenic factor 6, and myocyte enhancer factor 2C, were examined. Representative skeletal muscle-associated genes, including creatine kinase (muscle), myoglobin, titin, nebulin, and desmin, were also examined in the C, HFD, and HFD + VE groups. These analyses assessed whether the exercise-associated muscle-related transcriptional signature might be caused by substantial skeletal muscle contamination of the BAT samples.

### 2.7 Statistical analyses

Microarray data are presented as log2-transformed signal intensities, where appropriate. Statistical comparisons of gene expression among groups were performed in TAC using the eBayes ANOVA method (n = 4 per group). Nominal P values of <0.05 were used for exploratory transcript selection, and FDR-adjusted P values were additionally examined to account for genome-wide multiple testing. GO enrichment analyses were performed using DAVID. Nominal enrichment P values were used to rank and visualize GO biological process terms, and FDR-adjusted enrichment P values were also reported to assess multiple-testing-corrected enrichment.

## 3 Results

### 3.1 HFD induces transcriptomic changes in BAT

To comprehensively characterize HFD-associated transcriptomic changes in BAT, gene expression profiles were analyzed using the Clariom S Mouse Array. Using the prespecified exploratory criteria of an absolute fold change >2, a nominal P value < 0.05, and a mean log2 signal intensity ≥8 in the group with higher expression, 579 transcript clusters differed between the C and HFD groups (Fig. 1). GO enrichment analysis was subsequently performed separately for the 348 upregulated and 231 downregulated transcript clusters (Fig. 2). FDR-adjusted P values were additionally examined to account for genome-wide multiple testing. The most strongly altered transcripts included matrix metalloproteinase 12, glycoprotein nonmetastatic melanoma protein B, leptin, galectin 3, ATPase H+ transporting V0 subunit d2, cytochrome P450 family 2 subfamily E member 1, troponin T3, fast skeletal type (Tnnt3), cytochrome P450 family 2 subfamily B member 10, major urinary protein 12, and MFSD2A lysolipid transporter (Table 1).

**Fig. 1:**
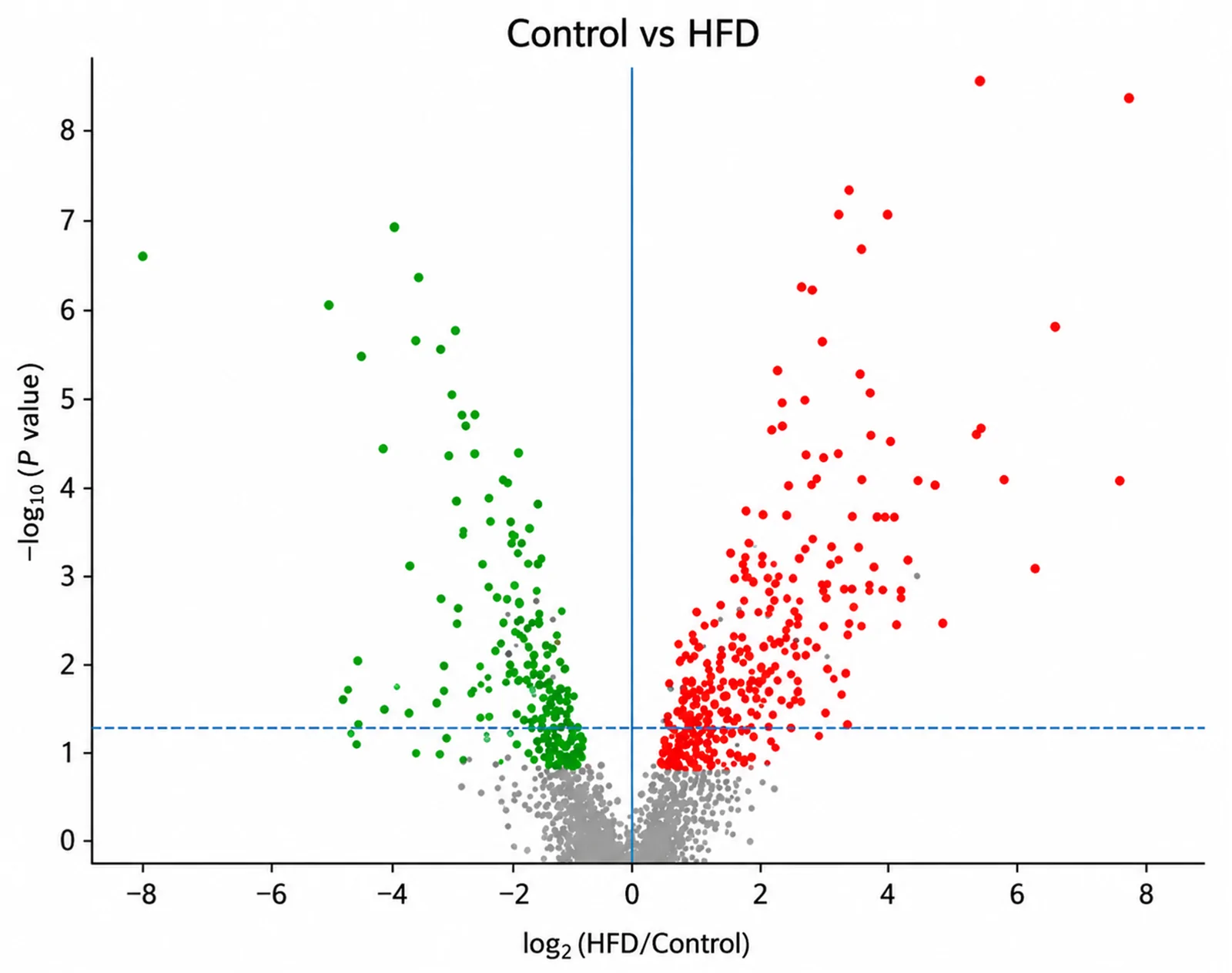
A volcano plot of transcript clusters in the control vs HFD groups. Each dot represents one transcript cluster. The x-axis represents log2 fold change (HFD vs control), and the y-axis represents -log10(nominal P value). Transcript clusters meeting the prespecified exploratory criteria of an absolute fold change >2, a nominal P value < 0.05, and a mean log2 signal intensity ≥8 in the group with higher expression are highlighted according to the direction of change; gray dots represent transcript clusters that did not meet these criteria.

**Fig. 2:**
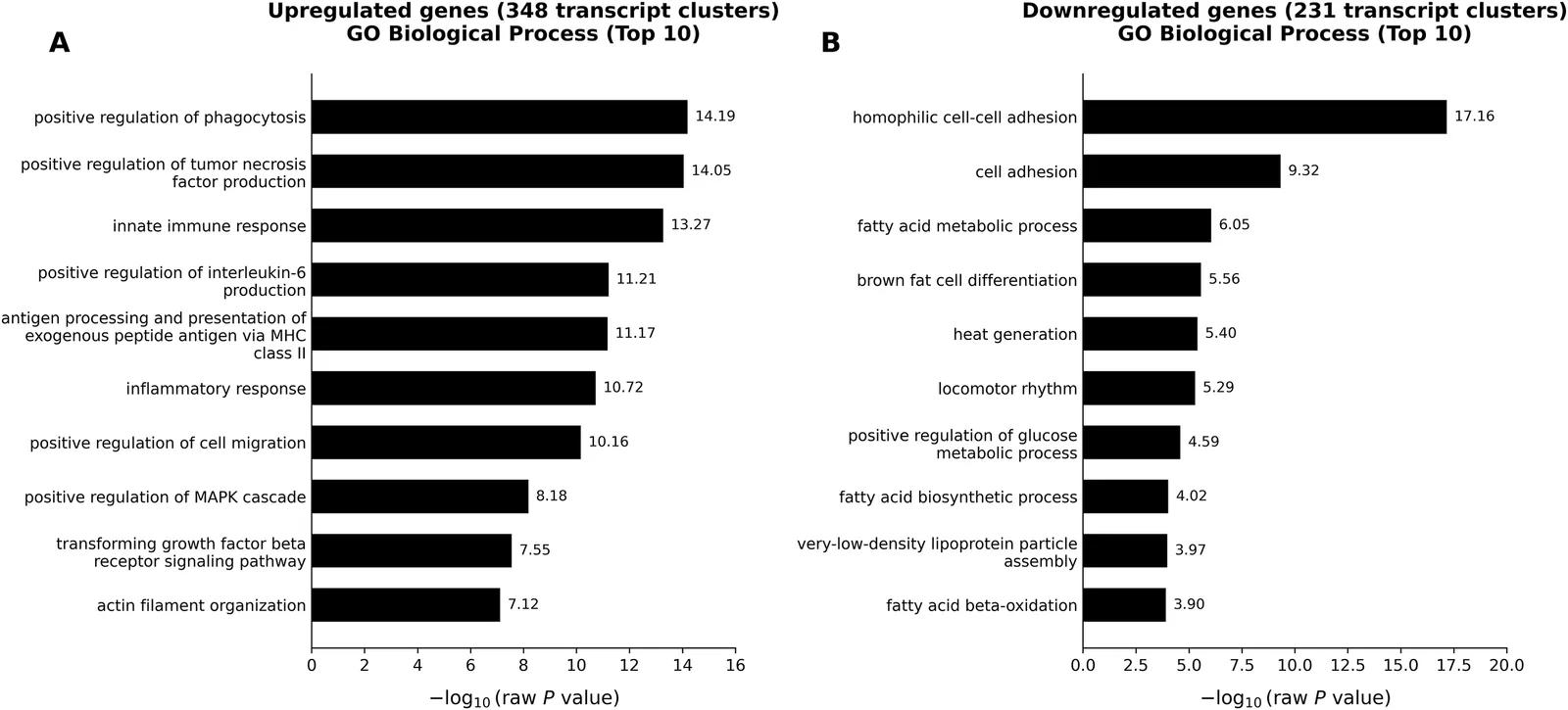
Gene ontology enrichment analysis of the genes that were significantly changed in brown adipose tissue by the HFD. Gene Ontology (GO) enrichment analysis was conducted on 579 transcript clusters (348 upregulated and 231 downregulated; *P* < 0.05 and mean log2 signal intensity ≥8 in the group with higher expression). (A) The top 10 GO biological process terms enriched in the genes upregulated by HFD. (B) The top 10 GO biological process terms enriched in the genes downregulated by HFD. GO terms were ranked by raw *P*-value. Enrichment significance is presented as -log10 (raw *P*-value).

**Table 1:** The top 10 upregulated and downregulated genes in the brown adipose tissue from mice on a high-fat diet.

Upregulated in HFD	Fold change (HFD/Cont)	*P*-value	Downregulated in HFD	Fold change (HFD/Cont)	*P*-value
*Mmp12*	248.94	1.09 × 10^−8^	*Cyp2e1*	0.009	3.11 × 10^−7^
*Gpnmb*	171.32	5.90 × 10^−5^	*Tnnt3*	0.021	0.0129
*Lep*	94.51	2.92 × 10^−6^	*Cyp2b10*	0.036	7.51 × 10^−7^
*Lgals3*	69.30	6.00 × 10^−4^	*Mup12*	0.042	0.0260
*Atp6v0d2*	58.33	7.22 × 10^−5^	*Mfsd2a*	0.042	0.0099
*Cd68*	48.03	2.47 × 10^−5^	*Mup13/18/16/14/17/2/15*	0.044	0.0072
*Mpeg1*	45.87	2.85 × 10^−5^	*Acta1*	0.045	0.0192
*Npr3*	32.45	7.30 × 10^−9^	*Cfd*	0.046	2.69 × 10^−6^
*Spp1*	30.26	0.0020	*Myh4*	0.046	0.0336
*Lyz2*	29.33	8.69 × 10^−5^	*Mup2/Mup1*	0.050	0.0048

*Some transcript clusters corresponded to multiple annotated gene symbols.

### 3.2 VE remodels biological processes in the BAT of HFD-fed mice

The mean daily running distance in the HFD + VE group was 4067 ± 711 m/day (mean ± SD, n = 4).

GO enrichment analysis was performed separately for the exploratory transcript sets showing lower or higher expression with VE in the HFD versus HFD + VE comparison. Of the 89 transcript clusters meeting the prespecified criteria (absolute fold change >2, nominal P < 0.05, and mean log2 signal intensity ≥8 in the group with higher expression), 74 showed lower expression and 15 showed higher expression with VE.

The 74 transcript clusters showing lower expression with VE were predominantly associated with immune- and inflammation-related biological processes (Fig. 3A). Several GO biological process terms remained significant after FDR correction for GO-term testing, including inflammatory response, receptor-mediated endocytosis, innate immune response, antigen processing and presentation of exogenous peptide antigen via MHC class II, and immune system process.

**Fig. 3:**
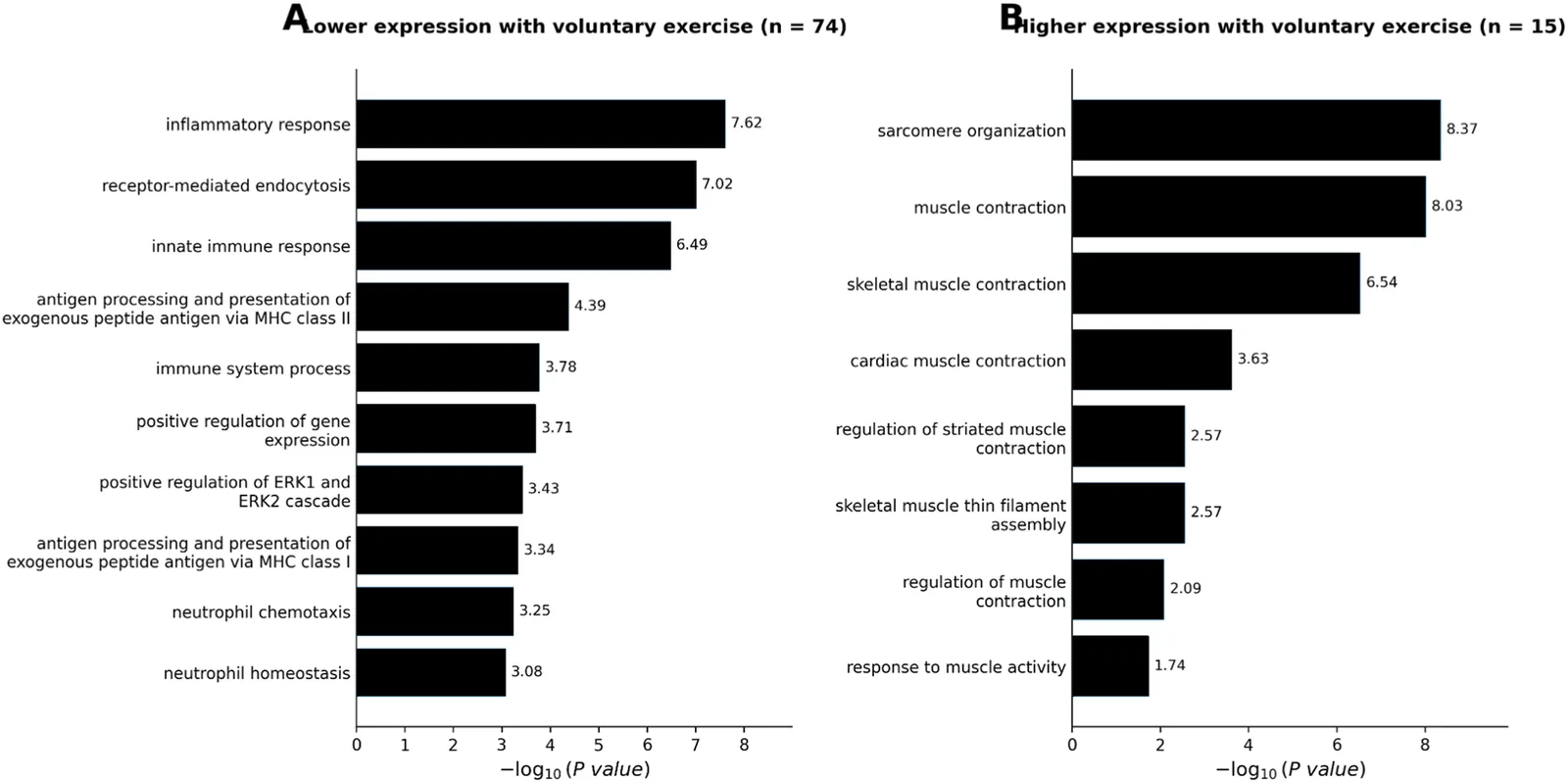
Gene ontology analysis of the biological processes enriched for in the genes altered by voluntary exercise in BAT from mice on the HFD. The 89 transcript clusters meeting the prespecified exploratory criteria (absolute fold change >2, nominal P < 0.05, and mean log2 signal intensity ≥8 in the group with higher expression) in the HFD versus HFD + voluntary exercise comparison were subjected to functional annotation using DAVID. (A) GO biological process terms enriched among the 74 transcript clusters showing lower expression with voluntary exercise. (B) GO biological process terms enriched among the 15 transcript clusters showing higher expression with voluntary exercise. GO terms were ranked by nominal P value, and FDR-adjusted enrichment P values were additionally examined to account for multiple GO-term testing. The x-axis indicates -log10(nominal P value).

The 15 transcript clusters showing higher expression with VE were predominantly enriched for muscle contraction- and sarcomere-related biological processes (Fig. 3B). Several GO biological process terms remained significant after FDR correction for GO-term testing, including sarcomere organization, muscle contraction, skeletal muscle contraction, cardiac muscle contraction, regulation of striated muscle contraction, and skeletal muscle thin filament assembly.

Together, these observations indicate that in BAT, VE is associated with the downregulation of immune- and inflammation-related genes, and the upregulation of a distinct set of muscle contraction-related genes.

At the individual-transcript level, however, none of the transcript clusters identified in the HFD versus HFD + VE comparison remained significant at FDR <0.05 after genome-wide multiple-testing correction. Accordingly, these exercise-responsive transcript clusters should be interpreted as exploratory candidates defined by the prespecified nominal P-value criteria rather than as genome-wide FDR-significant changes. Importantly, GO enrichment analysis of these exploratory transcript sets identified coordinated biological processes that remained significant after correction for multiple testing. Among the transcripts decreased with VE, FDR-significant GO terms included inflammatory response, receptor-mediated endocytosis, innate immune response, antigen processing and presentation via MHC class II, and immune system process. Among the transcripts increased with VE, FDR-significant terms included sarcomere organization, muscle contraction, skeletal muscle contraction, cardiac muscle contraction, regulation of striated muscle contraction, and skeletal muscle thin filament assembly. Thus, although the evidence for individual exercise-responsive transcripts did not reach genome-wide FDR significance, the pathway-level analysis supports coordinated enrichment of immune/inflammatory and muscle contraction-related transcriptional signatures.

Next, we identified HFD-responsive transcript clusters that also met the exploratory criteria in the HFD versus HFD + VE comparison and changed in the opposite direction. This analysis identified 66 transcript clusters: 60 were increased in HFD relative to C and decreased with VE, whereas six were decreased in HFD relative to C and increased with VE.

GO enrichment analysis of the six transcript clusters decreased by HFD and increased with VE identified muscle contraction-related processes (Fig. 4A). Skeletal muscle contraction remained significant after FDR correction for GO-term testing (FDR = 3.71 × 10−4), as did regulation of striated muscle contraction (FDR = 0.017) and regulation of muscle contraction (FDR = 0.0339). Sarcomere organization was nominally enriched but did not remain significant after FDR correction (nominal P = 0.00884, FDR = 0.0663).

**Fig. 4:**
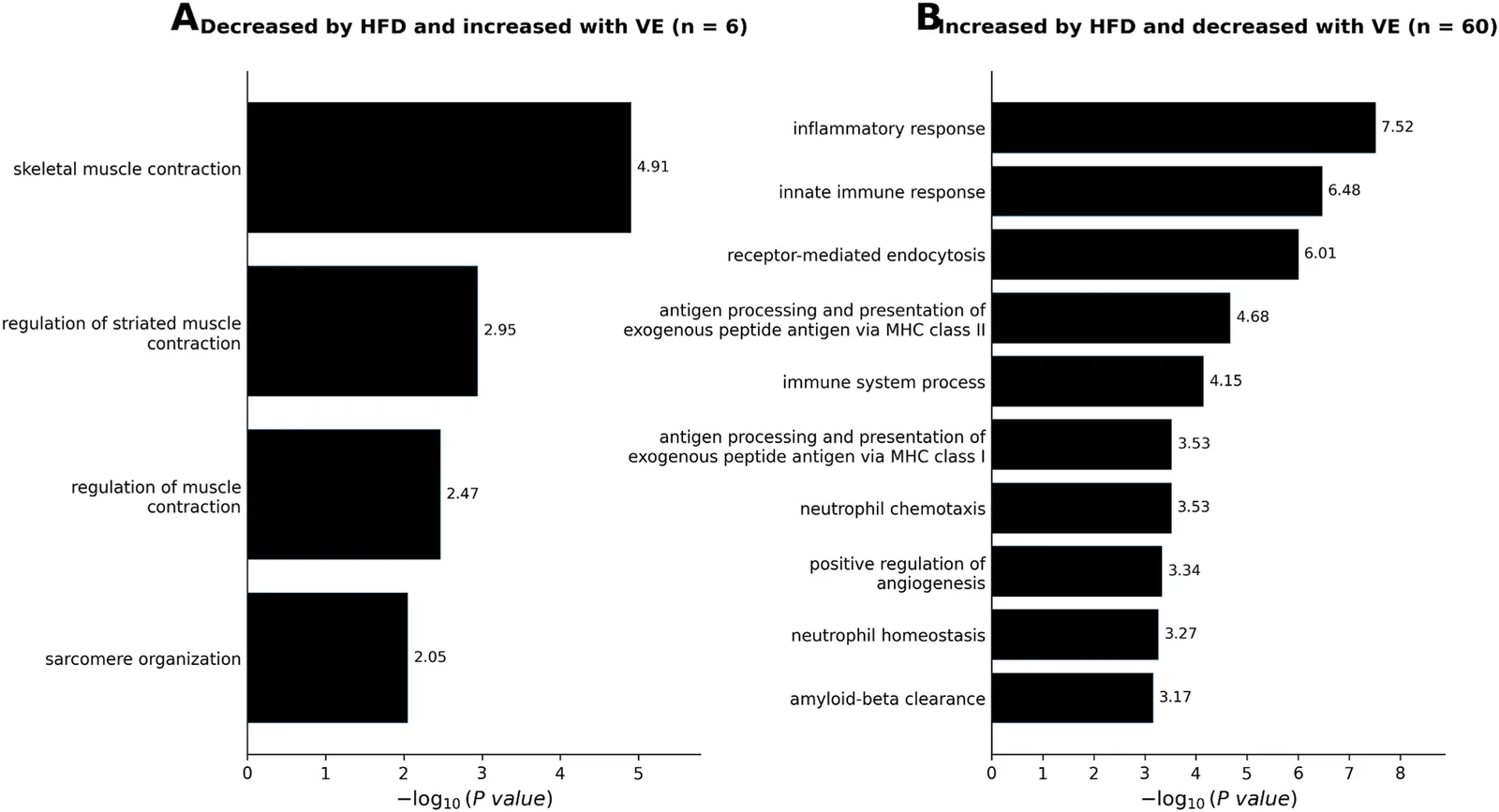
GO biological process enrichment analysis of HFD-responsive transcript clusters showing opposite-direction changes with voluntary exercise. Transcript clusters meeting the prespecified exploratory criteria (absolute fold change >2, nominal P < 0.05, and mean log2 signal intensity ≥8 in the group with higher expression) in both the C versus HFD and HFD versus HFD + voluntary exercise comparisons and changing in opposite directions were subjected to DAVID functional annotation. A total of 66 transcript clusters met these criteria. (A) GO biological process terms enriched among the six transcript clusters decreased by HFD and increased with voluntary exercise. (B) GO biological process terms enriched among the 60 transcript clusters increased by HFD and decreased with voluntary exercise. In panel A, skeletal muscle contraction, regulation of striated muscle contraction, and regulation of muscle contraction remained significant after FDR correction for GO-term testing. In panel B, multiple immune- and inflammation-related processes, including inflammatory response, innate immune response, receptor-mediated endocytosis, antigen processing and presentation via MHC class II, and immune system process, remained significant after FDR correction. GO terms were ranked by nominal P value; FDR-adjusted enrichment P values were additionally reported to account for multiple GO-term testing. The x-axis indicates -log10(nominal P value).

Conversely, GO enrichment analysis of the 60 transcript clusters increased by HFD and decreased with VE showed strong enrichment of immune- and inflammation-related biological processes (Fig. 4B). Terms that remained significant after FDR correction included inflammatory response (FDR = 2.21 × 10−5), innate immune response (FDR = 1.21 × 10−4), receptor-mediated endocytosis (FDR = 2.38 × 10−4), antigen processing and presentation of exogenous peptide antigen via MHC class II (FDR = 0.00381), and immune system process (FDR = 0.0102). Additional FDR-significant terms included antigen processing and presentation via MHC class I, neutrophil chemotaxis, positive regulation of angiogenesis, neutrophil homeostasis, and amyloid-beta clearance.

The recurrence of immune-related processes in both the direct HFD versus HFD + VE comparison and the 60-transcript opposite-direction set supports a coordinated VE-associated shift opposing part of the HFD-associated immune and inflammatory transcriptional response. The six-transcript set likewise showed an opposite-direction pattern in muscle contraction-related genes. Because these transcript clusters were selected using nominal P-value and fold-change criteria and individual HFD versus HFD + VE transcript clusters did not remain significant after genome-wide FDR correction, these findings should be interpreted as exploratory pathway-level evidence rather than proof of transcript-level restoration.

Together, these analyses identify coordinated transcriptomic patterns that changed in opposite directions with HFD and VE, particularly an immune/inflammatory signature among the 60 HFD-increased/VE-decreased transcript clusters and a restricted muscle contraction-related signature among the six HFD-decreased/VE-increased transcript clusters.

## 4 Discussion

This study comprehensively characterized the BAT transcriptome following a chronic HFD and long-term VE. HFD induced broad transcriptional remodeling characterized by immune- and inflammation-related changes and alterations in biological processes associated with cell adhesion, lipid metabolism, brown fat cell differentiation, and thermogenic function. VE was associated with transcriptional shifts in the opposite direction to a subset of HFD-associated changes. These findings are consistent with previous observations that HFD-induced obesity enhances immune and inflammatory gene expression while suppressing lipid metabolism- and oxidation-associated genes in BAT, and that some of these alterations are counteracted by aerobic exercise [14]. Notably, the present analysis identified 66 transcript clusters showing opposite-direction changes between the C versus HFD and HFD versus HFD + VE comparisons, including a prominent immune/inflammatory set and a restricted group associated with muscle contraction-related processes.

Although no individual transcript clusters remained significant after genome-wide FDR correction, several GO biological process terms identified by exploratory enrichment analysis showed significant enrichment after FDR correction for multiple GO-term testing. This pathway-level enrichment does not alter the absence of transcript-level FDR significance and should therefore be interpreted as exploratory evidence of coordinated changes in functionally related transcripts.

Classical brown adipocytes and skeletal muscle cells are developmentally related, and both lineages arise from Myf5-expressing progenitors [16]. Because interscapular BAT is anatomically located near skeletal muscles in mice, careful dissection is crucial for minimal skeletal muscle contamination. Although we cannot rule out a small contribution from contaminating skeletal muscles, several observations indicate that substantial contamination is not the primary cause of the muscle-related transcriptional changes. Canonical myogenic regulatory factors involved in skeletal muscle differentiation, including myogenic differentiation 1*,* myogenin*, Myf5,* myogenic factor 6*,* and myocyte enhancer factor 2C, were expressed at very low levels or did not differ significantly across the three groups. Similarly, representative skeletal muscle-associated genes, including creatine kinase (muscle)*,* myoglobin*,* titin*,* nebulin*,* and desmin, were not significantly altered across the groups (data not shown). Thus, the absence of coordinated changes in canonical myogenic regulatory factors and representative muscle markers argues against substantial skeletal muscle contamination as the major determinant of the observed transcriptional signature.

Interestingly, the six HFD-decreased/VE-increased transcript clusters comprised Acta1, Tnnt3, Tnni2, Casq1, Myh8, and Myot, representing a restricted set of genes associated with contractile machinery and Ca2+ handling rather than a broad myogenic differentiation program [[17], [18], [19], [20], [21], [22]]. This selective opposite-direction pattern raises the possibility that VE may influence a distinct contractile/Ca2+-related transcriptional program in BAT. Notably, Ca2+-dependent processes have been implicated in exercise-induced BAT adaptation. Fu et al. reported that aerobic exercise enhanced VEGF signaling in the BAT of mice with HFD-induced obesity and proposed a signaling cascade involving VEGF receptor activation, PLCγ, IP3/Ca2+ signaling, calcineurin/NFAT, and COX2 [13]. Although the genes identified in this study differ from the VEGF-COX2 pathway emphasized in that study, the opposite-direction changes in genes associated with Ca2+ handling and Ca2+-dependent contractile machinery provide an independent transcriptional observation that may converge on Ca2+-related processes. This is intriguing because intracellular Ca2+ signaling is closely linked to mitochondrial function and metabolic regulation in brown adipocytes [[23], [24], [25]].

However, this study's findings should be interpreted with caution. By themselves, changes in *Acta1, Tnnt3, Tnni2, Casq1, Myh8,* and *Myot* do not demonstrate Ca^2+^ signaling, and our whole-tissue transcriptomic analysis cannot establish the cellular origin of these genes. Nevertheless, together with previous evidence implicating VEGF–PLCγ–Ca^2+^-related signaling in exercise-induced BAT adaptation, our results raise the possibility that Ca2+-associated cellular processes may contribute to exercise-associated BAT remodeling. Direct assessment of Ca^2+^ dynamics and Ca^2+^-responsive signaling pathways is required to determine if this transcriptional signature has functional significance.

Another prominent feature of this study was the marked immune and inflammatory transcriptional response to the HFD and its modification by VE. The biological processes enriched among HFD-associated transcripts included immune and inflammatory pathways, while transcript clusters showing lower expression with VE were also strongly associated with inflammatory response, innate immune response, antigen processing and presentation, and leukocyte-related processes. This is consistent with previous findings by Fu et al., in which HFD-induced obesity increased immune- and inflammation-related gene expression in BAT and aerobic exercise exerted anti-inflammatory effects [13]. Importantly, the 60 transcript clusters that increased with HFD and changed in the opposite direction with VE were strongly enriched for immune-related biological processes, with inflammatory response, innate immune response, receptor-mediated endocytosis, antigen processing and presentation via MHC class II, and immune system process remaining significant after FDR correction for GO-term testing. This concordance supports a coordinated VE-associated transcriptional shift opposing part of the HFD-associated immune response, while not establishing transcript-level restoration to control values.

Obesity is accompanied by chronic, low-grade adipose tissue inflammation characterized by the recruitment and activation of immune cells, particularly macrophages [26,27]. Although inflammatory remodeling is most extensively studied in WAT, BAT is also susceptible to obesity-associated immune activation and local cellular microenvironment changes, which are consistent with the enrichment of phagocytosis, antigen presentation, inflammatory response, and immune cell-related processes observed in this study. Therefore, their attenuation by VE suggests that immune microenvironment improvement may be an important component of exercise-induced BAT adaptation.

However, our transcriptomic analysis cannot distinguish altered gene expression in brown adipocytes from changes in the abundance or activation state of immune cells and other stromal cells since it was performed on whole BAT. This limitation is particularly relevant to genes involved in MHC class II antigen presentation and leukocyte-related processes. Hence, the reduction in immune-related transcriptional signatures may reflect reduced immune cell infiltration, altered immune cell activation, resident cell transcriptional changes, or a combination of these effects, and histological and cell type-specific analyses are needed to distinguish between these possibilities.

The HFD was also associated with the suppression of the biological processes related to cell adhesion and BAT metabolic function. The GO terms that were most significantly enriched for in the HFD-downregulated genes included homophilic cell–cell adhesion, cell adhesion, fatty acid metabolic process, brown fat cell differentiation, heat generation, and fatty acid β-oxidation. Notably, the enrichment for cell adhesion-related processes included a transcript cluster annotated to multiple members of the clustered protocadherin family, particularly protocadherin gamma cluster members (Supplementary Table S1), which are involved in cell–cell recognition and interactions [28,29], although their specific physiological role in BAT is poorly understood. Therefore, the reduction of this protocadherin-associated transcript cluster suggests that obesity may alter cell–cell interactions or tissue organization within BAT, and these findings should not be interpreted as direct evidence of impaired cell adhesion in brown adipocytes. This possibility warrants further investigation. Along with the suppression of fatty acid metabolism, brown fat cell differentiation, heat generation, and fatty acid β-oxidation, these findings indicate that a chronic HFD disrupts multiple components of the differentiated and metabolically active BAT phenotype.

The exercise paradigm is an important difference between this study and previous work. The study by Fu et al. assessed four weeks of forced treadmill exercise after establishing HFD-induced obesity, while this study examined long-term voluntary wheel running while the HFD continued. Physiological adaptation can be substantially influenced by exercise modality and duration, and voluntary wheel running avoids some of the stress associated with forced treadmill exercise. However, both studies revealed the attenuation of obesity-associated inflammatory changes in BAT despite their methodological differences, indicating this response is a reproducible component of exercise-induced BAT remodeling. In contrast, the contractile/Ca^2+^-related transcriptional signature observed in this study may represent an adaptation associated with the long-term VE paradigm, although direct comparisons between exercise modalities are needed to establish this possibility.

An additional limitation is the absence of a control diet plus VE (C + VE) group. Therefore, exercise-specific transcriptional effects cannot be distinguished from VE-associated changes occurring under HFD conditions.

Another limitation is the absence of independent qPCR validation of selected transcriptomic changes because the RNA samples were exhausted during the microarray experiments. Therefore, the individual transcript-level findings should be regarded as exploratory and require independent validation in future studies.

Taken together, this study shows that chronic HFD was associated with broad transcriptional remodeling of BAT, including immune- and inflammation-related changes and alterations in cell adhesion-related, metabolic, and thermogenic transcriptional programs. Long-term VE was associated with transcriptomic changes that opposed a subset of HFD-associated changes, particularly in immune-related processes. Although no individual transcript cluster in the HFD versus HFD + VE comparison remained significant after genome-wide FDR correction, exploratory GO analysis identified coordinated immune/inflammatory and muscle contraction-related processes that remained enriched after FDR correction for GO-term testing. The restricted six-gene contractile/Ca2+-related transcriptional signature may represent a potentially interesting component of exercise-associated BAT remodeling (Fig. 5), but the transcriptomic data alone do not demonstrate altered Ca2+ signaling. Together with previous evidence implicating VEGF-PLCγ-Ca2+-related signaling in exercise-induced BAT adaptation, these findings support further investigation of Ca2+-associated processes in BAT. The cellular origin and functional consequences of these changes remain unclear because this study is based on whole-tissue transcriptomic analysis. Studies integrating cell type-specific analyses with functional assessment of immune cell composition, BAT metabolic function, and Ca2+-related signaling are needed to define the mechanisms of exercise-induced BAT remodeling.

**Fig. 5:**
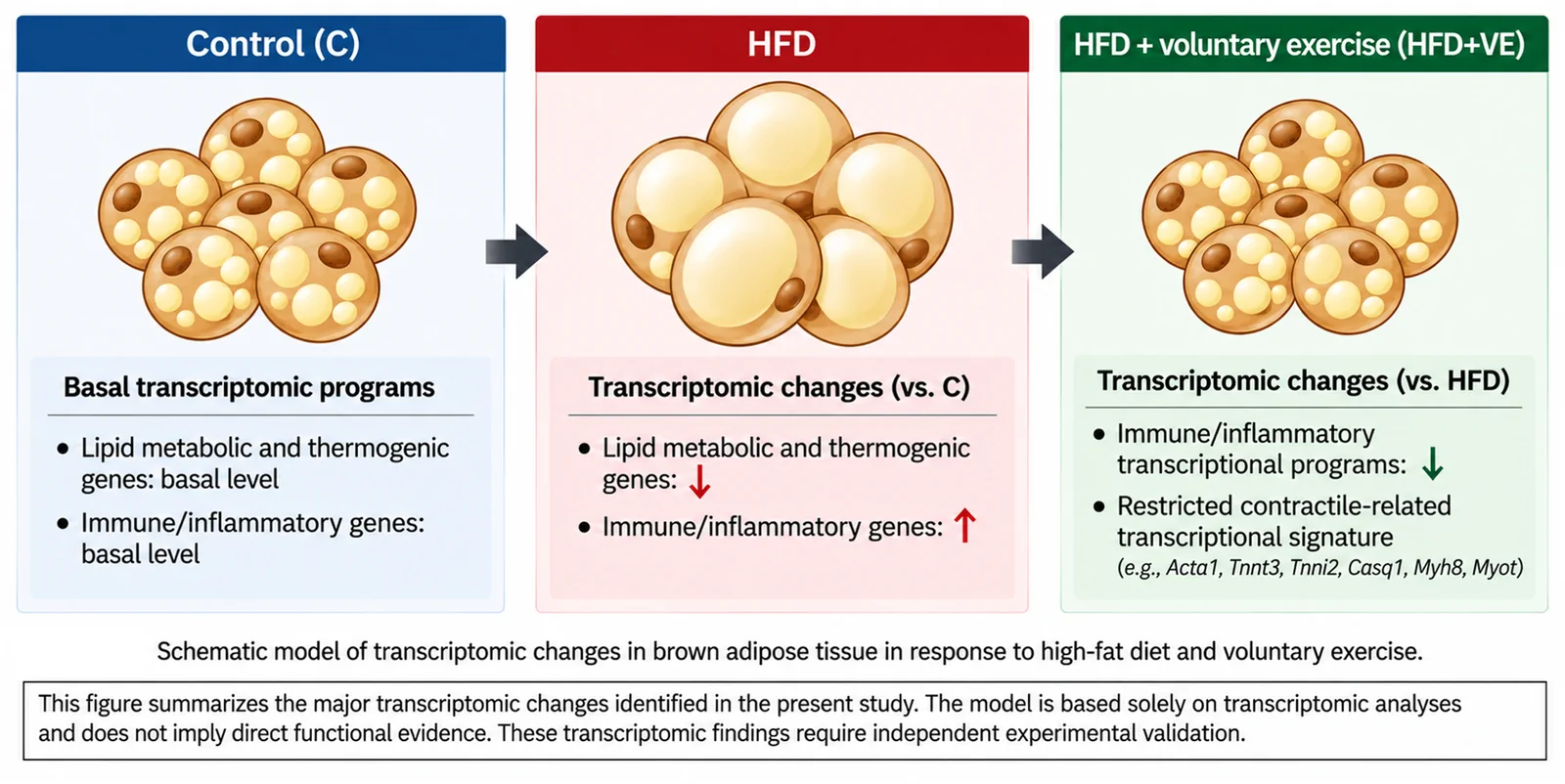
Schematic summary of HFD- and voluntary exercise-associated transcriptomic changes in brown adipose tissue. Chronic HFD was associated with decreased lipid metabolic and thermogenic transcriptional programs and increased immune/inflammatory transcriptional programs. Voluntary exercise was associated with decreased immune/inflammatory transcriptional programs relative to HFD and with a restricted muscle contraction-related transcriptional signature. The figure summarizes the major transcriptomic patterns identified in the present study. These findings are based solely on transcriptomic analyses and do not imply direct functional evidence. Independent experimental validation is required. The schematic illustration was prepared with the assistance of ChatGPT (OpenAI), and its scientific content and final design were reviewed and approved by the authors. (For interpretation of the references to colour in this figure legend, the reader is referred to the Web version of this article.)

## CRediT authorship contribution statement

**Takuya Sakurai:** Writing – review & editing, Writing – original draft, Methodology, Investigation, Formal analysis, Data curation, Conceptualization. **Eriko Nozaki:** Investigation. **Tetsuya Iwawa:** Writing – review & editing, Investigation.

## Ethics declaration

This study was conducted in accordance with the ARRIVE (Animal Research: Reporting of In Vivo Experiments) guidelines. This study was approved by the Experimental Animal Ethics Committee of Kyorin University School of Medicine. (Approval No. 76)

## Availability of data and materials

The microarray data generated during the current study will be deposited in the Gene Expression Omnibus (GEO) database prior to acceptance of the manuscript. All other data generated and/or analyzed during this study are available from the corresponding author on reasonable request.

## Ethics approval and consent to participate

All animal experiments were approved by the Experimental Animal Ethics Committee of Kyorin University School of Medicine (Approval No. 76, approved on April 1, 2018) and were conducted in accordance with the institutional guidelines.

## Declaration of generative AI and AI-assisted technologies in the manuscript preparation process

During the preparation of this work, the authors used ChatGPT (OpenAI) to assist with language editing, manuscript organization, improvement of readability, and the preparation and refinement of the schematic illustration in Fig. 5. After using this tool, the authors reviewed and edited the content as needed and take full responsibility for the content of the publication.

## Funding

This work was partially supported by 10.13039/501100001691JSPS KAKENHI Grant Number 25K14616.

## Declaration of competing interest

The authors declare that they have no known competing financial interests or personal relationships that could have appeared to influence the work reported in this paper.
