# Supplementary material for: Long-term voluntary exercise partially reverses obesity-associated transcriptomic remodeling in brown adipose tissue
Source: Metabol Open. 2026 Oct 6;32:100504. doi: 10.1016/j.metop.2026.100504 (PMC13676147; doi:10.1016/j.metop.2026.100504)
Supplement: Supplementary file 1 [file mmc1.docx]

**Supplementary Table S1**

**Protocadherin transcript cluster significantly reduced in brown adipose tissue by HFD**

| **Transcript cluster ID** | **Gene symbol(s)** | **Cont Avg (log2)** | **HFD Avg (log2)** | **Fold change** | **P value** | **FDR P value** |
| --- | --- | --- | --- | --- | --- | --- |
| TC1800001741.mm.2 | Pcdhgb1; Pcdhgb2; Pcdhgb4; Pcdhgb5; Pcdhgb6; Pcdhgb7; Pcdhgb8; Pcdhgc3; Pcdhgc4; Pcdhgc5; Pcdhga1; Pcdhga2; Pcdhga3; Pcdhga4; Pcdhga5; Pcdhga6; Pcdhga7; Pcdhga8; Pcdhga9; Pcdhga10; Pcdhga11; Pcdhga12 | 10.56 | 9.77 | 1.73 | 0.0472 | 1.0 |

**Note.** Values are derived from the Clariom S Mouse microarray comparison of control (Cont) and high-fat diet (HFD) groups. Positive fold-change values in the source output indicate higher expression in Cont than HFD. The transcript cluster TC1800001741.mm.2 maps to multiple clustered protocadherin gamma genes; therefore, the listed gene symbols should not be interpreted as independently quantified transcripts. The cluster met the study's raw P-value criterion (P < 0.05), whereas the FDR-adjusted P value was not significant.
